# Diversity of Biogenic Nanoparticles Obtained by the Fungi-Mediated Synthesis: A Review

**DOI:** 10.3390/biomimetics8010001

**Published:** 2022-12-20

**Authors:** Ekaterina A. Loshchinina, Elena P. Vetchinkina, Maria A. Kupryashina

**Affiliations:** Laboratory of Microbiology, Institute of Biochemistry and Physiology of Plants and Microorganisms, Saratov Scientific Centre of the Russian Academy of Sciences (IBPPM RAS), 13 Prospekt Entuziastov, 410049 Saratov, Russia

**Keywords:** biogenic nanoparticles, green synthesis, metals and metalloids, synthesis conditions, nanoparticle characteristics

## Abstract

Fungi are very promising biological objects for the green synthesis of nanoparticles. Biogenic synthesis of nanoparticles using different mycological cultures and substances obtained from them is a promising, easy and environmentally friendly method. By varying the synthesis conditions, the same culture can be used to produce nanoparticles with different sizes, shapes, stability in colloids and, therefore, different biological activity. Fungi are capable of producing a wide range of biologically active compounds and have a powerful enzymatic system that allows them to form nanoparticles of various chemical elements. This review attempts to summarize and provide a comparative analysis of the currently accumulated data, including, among others, our research group’s works, on the variety of the characteristics of the nanoparticles produced by various fungal species, their mycelium, fruiting bodies, extracts and purified fungal metabolites.

## 1. Introduction

The most important task of modern nanotechnology is the development of reliable and efficient techniques allowing one to produce monodisperse nanoparticles with needed parameters. Nanoparticle production methods can be divided into physical, chemical and biological (or bio-assisted) [1,2], as well as combined methods putting together biological materials and physical influences, such as microwave radiation [3,4]. The chemical and physical methods traditionally used to produce nanoparticles allow large quantities of particles to be synthesized in a short time, but they are often expensive and difficult to perform; the issue of their environmental safety is a big problem as well. Therefore, in recent years, there has been an increasing interest in green nanotechnology and the biological synthesis of nanoparticles [5,6,7,8]. The introduction of green synthesis techniques can reduce the negative impact of nanotechnology on the environment by using less toxic reagents and reducing the risks of secondary pollution. Furthermore, nanoparticles produced via biosynthesis may have higher stability and biocompatibility and lower toxicity owing to their coating with biogenic surfactants or capping agents [9,10,11].

The ability to form nanoparticles has been found in all groups of organisms. Numerous studies have shown that plants [12,13,14,15], animals [10,16,17], bacteria [16,18], fungi [11,19,20,21], actinomycetes [22,23], algae [14,16,24,25], lichens [26] and viruses [10] can be successfully used to produce nanoparticles. Along with living cultures, their biomass, cell fractions, extracts, metabolites and spent media can also be used in green nanosynthesis [6,11,12,14,27,28], as well as various plant and animal food products [29,30,31,32] and organic industrial wastes [33,34,35]. Fungal cultures of different taxonomic groups are very promising biological objects for the green synthesis of nanoparticles [11,20,36]. The advantage of fungi in comparison to other organisms is their ability to produce a wide range of active protein molecules, convert ions of heavy metals and other trace elements into less toxic forms under the action of their enzymes and accumulate them in large quantities both within their mycelium and extracellularly. Therefore, fungi-mediated nanosynthesis has been increasingly studied in the past two decades. In fungal cultures, the ability to form a wide range of nanoparticles of different chemical compositions was found, including metals, metalloids, metal oxides and sulfides and other compounds, as well as composite nanoparticles [19,36,37].

All biological methods of nanosynthesis can be divided into two main types. In the first one, living cultures are used directly to serve as “nanofactories“, forming nanoparticles in vivo and accumulating them in their cells, on their cell surface or in the medium. Fungal biomass separated from the culture liquid can also be used. The disadvantage of this method is the need to separate the obtained nanoparticles from the bio-object’s cells; moreover, the process of biosynthesis by growing cultures may take a long time. The level of the precursor may also be a limiting factor, because high concentrations of metals and other compounds used to biosynthesize nanoparticles inhibit the growth processes. Another option for green nanoparticle synthesis is the use of various substances derived from bio-objects, such as culture liquids, intracellular extracts, protein fractions or individual fungal metabolites. The use of such techniques greatly facilitates the process of nanosynthesis, as there is no need to destroy the producing organism’s cells and separate nanoparticles from them.

The physico-chemical properties of nanoparticles are closely related not only to their chemical composition and crystal structure but also to their size and morphology, including particle physical shape, surface topography and the presence of pores and cavities [38,39]. It is also important that the synthesized nanoparticles are homogeneous in size and shape and resistant to aggregation in suspensions. The properties of biogenic nanoparticles have been found to depend on the species and strain of the microorganism, the extracts and metabolites used, the precursor compound and its concentration, media composition, stirring rate, incubation time, temperature, pH and other conditions, the varying of which can control nanoparticle formation [40,41,42]. The characteristics of the resulting nanoparticles determine their future applications. In this regard, an important challenge in nanobiotechnology is to develop methods allowing one to produce nanoparticles with better control over their size, shape and other properties. However, not enough attention has been paid to the study of the influence of the synthesis conditions on the properties of biogenic nanoparticles. In particular, there are still few comparative studies on the mycosynthesis of nanoparticles with different characteristics using the same fungal species but under different conditions.

In this review, we tried to summarize the currently available data on the variety of characteristics of the nanoparticles produced by various fungal species, their mycelium, fruit bodies, extracts and purified fungal metabolites. We paid special attention to the nanoparticles of the elements that currently remain understudied and insufficiently covered in reviews.

## 2. Fungi-Mediated Synthesis of Nanoparticles

### 2.1. Mycosynthesis of Silver Nanoparticles

Owing to their unique features, silver nanoparticles (AgNPs) have many applications in various fields of medicine and engineering. The area of their applications includes electronic components, biomedical devices, textile engineering, cosmetics, agricultural engineering and many others [43,44]. AgNPs have been found to have a wide range of biological activities, including antibacterial, antifungal, antiviral, antitumor, hepatoprotective and hypotensive properties, which is why they are actively used for therapeutic purposes [45,46].

To date, the biological synthesis of AgNPs in fungi has been the most extensively studied of all elements. The ability to produce nanosilver has been detected in more than 120 species of fungi from different taxa, including Ascomycota (*Alternaria* [47], *Aspergillus* [48,49], *Beauveria* [50], *Bionectria* [51], *Botryodiplodia* [52], *Chrysosporium* [53], *Cladosporium* [54], *Colletotrichum* [55], *Epicoccum* [56], *Fusarium* [57,58], *Geotricum* [59], *Guignardia* [60], *Helvella* [61], *Hormoconis* [62], *Humicola* [63], *Macrophomina* [64], *Neurospora* [65], *Paecilomyces* [66], *Penicillium* [67,68,69,70,71,72], *Pestalotia* [73], *Phoma* [74,75], *Picoa* [76], *Saccharomyces* [77], *Sclerotinia* [78], *Scopulariopsis* [79], *Talaromyces* [80], *Tirmania* [81], *Trichoderma* [42,48,82,83,84], *Verticillium* [85], *Yarrowia* [86]), Mucoromycota (*Rhizopus* [87]) and Basidiomycota (*Agaricus* [41,88,89,90,91], *Auricularia* [92], *Bjerkandera* [93], *Boletus* [94], *Calocybe* [95], *Coriolus* [94], *Cryptococcus* [96], *Flammulina* [97,98], *Fomes* [99], *Fomitopsis* [100], *Ganoderma* [41,82,101,102], *Grifola* [41], *Hypsizygus* [103], *Inonotus* [104], *Lactarius* [105], *Laxitextum* [106], *Lentinus* [41,107], *Microporus* [108], *Phaenerochaete* [109], *Phellinus* [88], *Piriformospora* [110], *Pleurotus* [41,111,112,113,114], *Pycnoporus* [115], *Rhodotorula* [116], *Schizophylluum* [117], *Trametes* [118], *Tricholoma* [119], *Volvariella* [120]). Basidiomycetes are of particular interest as promising bio-objects for nanoparticle fabrication. Most of the basidiomycetes studied for mycosynthesis belong to edible and medicinal mushrooms, many of which are grown in artificial culture. These fungi produce a wide range of biologically active molecules, which not only can act as capping and stabilizing agents but also have anticancer, anti-inflammatory, antioxidant and antimicrobial activities themselves, allowing the production of nanoparticles with complex biomedical properties.

The number of research papers on nanosilver mycosynthesis includes many dozens and is constantly growing. In recent years, there have been several reviews detailing the production of biogenic nanoparticles of this element using fungal cultures [45,121,122]. Therefore, below we will focus on some of the most recent publications in the past five years (Table 1).

As can be seen from the table, AgNPs are commonly spherical in shape; irregular, oval, cubic, triangular, polygonal and other shapes are less common. Extracts from fruit bodies and mycelium are the most frequently used biological material for AgNP mycosynthesis, while culture liquids, biomass, living cultures and fungal metabolites of different purity (including enzymes, polysaccharides and phenolic compounds) are less commonly used.

A number of researchers have screened fungal cultures to find the most promising ones for AgNP biofabrication. For example, Qu et al. studied 10 *Trichoderma* species and found that AgNPs obtained using different species differed in the degree of antimicrobial activity [42]. Other researchers found that among nine different fungi isolated from metal-rich sites, a strain of *Penicillium janthinellum* exhibited maximum metal tolerance capacity and AgNP-synthesizing ability [69].

The shape, size, homogeneity and stability of nanoparticles are influenced by the process conditions, the optimization of which can improve the quality of the obtained particles. For example, Mohanta et al. used various ratios of a *Ganoderma sessiliforme* mushroom extract to AgNO_3_ for AgNP synthesis [101]. At a 0.5:10 ratio, nanoparticles formed very slowly; at a 1.5:10 ratio, the reaction was very rapid but nanoparticles formed large aggregates. The 1:10 ratio was optimal and allowed the authors to obtain nanoparticles with an average size of 45 nm with antimicrobial and antioxidant activity. In another study, the utilization of a *Ganoderma lucidum* fruit body extract was scrutinized under different operational conditions including the AgNO_3_:extract ratio, reaction time and temperature to establish an effective myconanosynthesis method with a high yield rate and nanoparticle stabilization [125]. Vetchinkina et al. studied the effect of the *Lentinus edodes* culture age and stage of ontogenesis on the biogenic AgNP synthesis using culture liquids of different ages [41] and extracts obtained from the different morphological structures of *L. edodes* [128]. Parametric optimization, including the concentration of AgNO_3_, fungal biomass, ratio of cell filtrate to AgNO_3_, pH, reaction time and presence of light, was performed for the rapid synthesis of silver nanoparticles by *Penicillium polonicum* [72]. For *Trichoderma harzianum* and *Ganoderma sessile,* different methods of mycelial extraction for silver mycosynthesis were compared [82]. The extract containing intracellular components of fungal strains was obtained from a mechanically disrupted mycelium, while for the extract containing extracellular components of fungal strains, the biomass was extracted without disruption. The second method produced smaller particles.

A number of researchers have studied the effect of various additional external physical influences on the fungi-mediated nanoparticle formation and developed combined methods of myconanosynthesis to improve AgNP characteristics. For example, UV radiation enhanced the characteristics of AgNPs obtained with an *Agaricus bisporus* pilei extract [89]. Microwave irritation enhanced the properties of AgNPs synthesized with the use of a *Pleurotus sajor-caju* fruit body extract [113]. AgNPs were synthesized from *Pleurotus florida* fruit body extracts using different electro-magnetic radiations, microwaves, visible light and UV rays [111]. Microwave irradiation led to the synthesis of monodisperse AgNPs of 10 nm size within 150 s of exposure, whereas visible light and UV radiation led to the synthesis of polydisperse AgNPs with inconsistent dimensions.

Numerous studies have shown that mycosynthesized AgNPs have antibacterial, antifungal, anticancer, antioxidant, larvicidal and other properties, and the same nanoparticles can exhibit a wide range of biological activities. For example, silver nanospheres obtained by using *Flammulina velutipes* had bactericidal, fungicidal, anti-Alzheimer, anticancer, antioxidant and anti-diabetic activities, as well as good biocompatibility against human red blood cells [97]. Silver nanospheres produced using *Aspergillus niger* and *Trichoderma longibrachiatum* xylanases exhibited antibacterial, antifungal, antioxidant, anticoagulant, thrombolytic and dye-degrading activities [48]. All these properties offer great prospects for biomedical applications of mycogenic AgNPs.

### 2.2. Mycosynthesis of Gold Nanoparticles

Gold nanoparticles (AuNPs) have attracted attention owing to their unique optical, electronic, thermal, chemical and biological properties. They have been used in chemical and biological sensing, bio-imaging, nonlinear optics, catalysis, targeted drug delivery, gene delivery and as antimicrobial and antioxidant agents, as well as in cancer, Alzheimer’s, cardiovascular and infectious disease therapy [129,130,131]. In the past two decades, the biological synthesis of gold nanoparticles by fungi has been studied almost as extensively as that of silver. The ability to form AuNPs has been found in dozens of micro- and macromycete species. The table shows the AuNP mycosynthesis data published in the past five years (Table 2).

As with silver, the ability to biosynthesize gold nanoparticles has been studied mainly in two groups of fungi—ascomycetes and basidiomycetes. Mycogenic AuNPs most often have a spherical shape, but triangular, hexagonal, cubic, irregular and other shapes were also found.

Needle- and flower-like nanostructures with a spindle shape were obtained using *Fusarium solani* biomass extract [140]. Spherical and hexagonal particles 22–30 nm in size were mycosynthesized with the use of *Fusarium oxysporum* cultural liquid [139].

Spherical, pentagonal and hexagonal nanoparticles (5–30 nm) were obtained with *Trichoderma hamatum* mycelial extract [149]. The authors optimized the conditions for the synthesis of AuNPs with the smallest size using *T. hamatum*. Nanoparticles biosynthesized using *T. harzianum* mycelial biomass had a nanometric size distribution below 30 nanometers and a spherical shape [150].

AuNPs of variable shapes with considerable antibacterial, antioxidant and antimitotic activities were obtained with an *Alternaria* spp. extract [135]. Gold nanospheres (10–100 nm) with antibacterial and antifungal properties were obtained using *Phoma* sp. mycelial biomass [148]. Cubic AuNPs with strong antimicrobial, cytotoxic and antioxidant activity were synthesized using a *Morchella esculenta* fruit body extract [147].

Molnár et al. studied AuNP mycosynthesis by 29 thermophilic fungi and compared the results of three different approaches for the synthesis of gold nanoparticles using the extracellular fraction, the autolysate or the intracellular fraction of the fungi [152]. They observed the formation of nanoparticles with different sizes (ranging between 6 nm and 40 nm) and size distributions depending on the fungal strain and experimental conditions.

Vetchinkina et al. studied AuNP mycosynthesis by *A. bisporus* and *Agaricus arvensis* cultures [41,153]. The use of live cultures, culture liquids and mycelial extracts resulted in the formation of nanoparticles of different sizes and shapes. Nanospheres were formed with living cultures and culture liquids, while irregularly spherical particles in the case of *A. bisporus* and various shapes with *A. arvensis* were formed using intracellular mycelial extracts.

An extract from the *A. bisporus* fruit body was prepared and utilized as a reducing and stabilizing agent toward a green synthesis of AuNPs [134]. The different parameters such as the precursor concentration, precursor:extract ratio, pH, temperature, reaction mode and reaction time were optimized for the mycosynthesis of AuNPs. The synthesized gold nanospheres (10–50 nm) significantly inhibited the growth of clinically important pathogenic Gram-positive and Gram-negative bacteria and pathogenic fungi. AuNPs with a dye-degrading activity obtained by Dheyab et al. using an *A. bisporus* fruit body extract were oval, spherical, drum-like, hexagonal and triangular (average size of 53 nm) [133]. An *A. bisporus* mushroom extract was also used to synthesize gold nanospheres through a hydrothermal process (at a pressure of 15 psi and a temperature of 121°C for 15 min) [132]. The optimal conditions for the maximum nanoparticle concentration and stability were selected.

Face-centered cubic nanocrystals with dye-reducing properties were synthesized using phenolic compounds isolated from *Ganoderma applanatum* [141]. Anticancer AuNPs biofabricated using a *G. lucidum* fruit body extract exhibited shapes such as spherical, oval and irregular, and their size ranged between 1 and 100 nm [142].

AuNPs synthesized using *G. lucidum* living cultures, as well as cultural liquid, were spherical, while the use of *G. lucidum* mycelial extract resulted in spherical, hexagonal, tetragonal and triangular particle formation [41]. The same results were obtained for *Grifola frondosa* and *Pleurotus ostreatus* cultures as well [41].

Chaturvedi et al. combined AuNP synthesis with the use of a *P. sajor-caju* fruit body extract followed by microwave irritation to further enhance the effects of fabricated gold nanospheres [113].

Vetchinkina et al. studied AuNP mycosynthesis by the *L. edodes* culture [41]. Living cultures formed nanospheres of 5–50 nm; smaller nanospheres were formed by the incubation of culture liquid with HAuCl_4_, and spherical, hexagonal, tetragonal and triangular particles of various sizes were formed with mycelial extract. Nanoparticles different in shape and size were synthesized using enzymes isolated and purified from the *L. edodes* mycelium. Spherical nanoparticles (2–20 nm) were obtained using intracellular Mn-peroxidase, and particles forming with the use of intracellular laccases and tyrosinases were bigger and irregularly spherical, triangular and tetrahedral in shape. When AuNPs were made with extracts from different morphogenetic stages of *L. edodes* and *G. lucidum*, their size, shape and degree of aggregation differed between the morphological structures involved [128]. The cytotoxicity of the AuNPs was negligible in a broad concentration range.

Other researchers used an *L. edodes* fruit body extract to produce AuNPs of various shapes [145].

Basu et al. obtained variously shaped gold nanoparticles using a *Tricholoma crassum* mycelial extract [151]. They showed that particle size could be altered by changing synthesis parameters such as temperature and substrate and precursor concentrations. A mixture of triangular, spherical and irregular shapes with an average size of 74.32 nm was fabricated using a *Flammulina velutipes* fruit body extract [138]. A chaga (*Inonotus obliquus*) medicinal mushroom extract induced the formation of mostly spherical AuNPs with a size below 20 nm [143]. These AuNPs are promising dual-modal (chemo-photothermal) therapeutic candidates for anticancer applications. The production of AuNPs by a *Coprinus comatus* fruit body extract and the effect of UV irradiation at different times on nanoparticle size were investigated [137]. Gold nanospheres were also obtained using fruit body extracts of *Cantharellus* sp. (average particle size of 60.6 nm) [136] and *Laetiporus versisporus* (average particle size of 10 nm) [144].

### 2.3. Mycosynthesis of Platinum Nanoparticles

Platinum nanoparticles (PtNPs) are of great interest in various fields of engineering and biomedicine owing to their unique physico-chemical (catalytic, magnetic and optical) and biological (antimicrobial, antioxidant, anticancer) properties [154,155,156]. The mycosynthesis of PtNPs is much less studied, as compared to that of silver and gold. To date, the ability to form nanoparticles of this noble metal has been detected in several Ascomycota species (Table 3).

PtNP biosynthesis has been best studied in *F. oxysporum*. Riddin et al. showed that the mycelial biomass of *F. oxysporum* is capable of producing nanoparticles of various shapes (hexagons, pentagons, circles, squares, rectangles) and sizes (10–100 nm) and determined the optimal conditions (pH, temperature and concentration of the precursor compound H_2_PtCl_6_) for maximum nanoparticle yield [158]. Nanoparticles were formed both extracellularly and intracellularly as well as on the hyphae surface, but only the extracellular production of nanoparticles proved to be statistically significant. In further studies [161], a hydrogenase with Pt(IV)-reductase activity was isolated from this strain of *F. oxysporum*. It was shown that the bioreduction of platinum salt by hydrogenase takes place by a passive process and not an active one as previously understood. PtNPs formed by cell-free mycelial extract and purified hydrogenase differed in size and shape. The particles formed with the extract were irregular in shape, with an average nanoparticle size of 30–40 nm. Circular, triangular, pentagonal and hexagonal nanoparticles, often appearing as nanoplates, with a mean size range of 40–60 nm, were formed using the enzyme. It was found that the oxidation state of the platinum salt also plays an important role in nanoparticle formation [160]. When PtCl_2_ was used as a precursor, large (100–180 nm) nanoparticles of predominantly rectangular and triangular shape forming aggregates were biosynthesized with *F. oxysporum* hydrogenase. Bioreduction of H_2_PtCl_6_ produced spherical monodisperse nanoparticles varying in size with the mean nanoparticle size between 100 and 140 nm.

Syed and Ahmad were able to produce spherical PtNPs with a diameter of 15–30 nm using *F. oxysporum* mycelial biomass [161]. The particles were formed extracellularly and were capped by natural proteins secreted by the fungus and therefore did not require the addition of stabilizing agents. Gupta and Chundawat obtained face-centered cubic nanoparticles with an average size of 25 nm with antimicrobial and photocatalytic activity using *F. oxysporum* filtrate [162]. 

The use of *Penicillium chrysogenum* culture filtrate made it possible to obtain highly dispersed non-aggregating platinum nanospheres (5–40 nm) [164]. Another ascomycete in which the ability to synthesize platinum nanoparticles has been found is *Neurospora crassa* [163]. Incubation of mycelial biomass with H_2_PtCl_6_ produced extracellular PtNPs (4–35 nm in diameter) and spherical nanoaggregates (20–110 nm in diameter). Using a mycelial extract from the same fungi, round single-crystal nanoagglomerates with diameters of 17 to 76 nm were obtained, containing individual single crystals of approximately 2–3 nm in diameter. Nanoplatinum was also obtained using the culture filtrate of the phytopathogenic fungus *Alternaria alternata* [157]. The particles were found to be irregular in shape presenting an overall quasi-spherical, rectangular, tetrahedral and hexagonal as well as polygonal morphology. Their size varied in the range of 50–315 nm with an average size of 135 nm.

Borse et al. investigated the production of platinum nanospheres using cell-free extract of *Saccharomyces boulardii* yeast biomass and the effect of parameters such as the concentration of H_2_PtCl_6_, temperature, pH, reaction time and cell concentration [165]. A cell mass concentration of 500 mg/ mLin, the presence of 0.5 mM chloroplatinic acid at 35 °C, pH 7 and 200 rpm for 36 h showed maximum PtNP synthesis. Under these conditions, platinum nanospheres (80–150 nm) with anticancer activity were formed. It was also shown that nanoparticles were formed intracellularly when whole yeast cells were incubated with H_2_PtCl_6_.

### 2.4. Mycosynthesis of Palladium Nanoparticles

The ability to biofabricate nanoparticles of another platinum-group noble metal, palladium, has been also discovered in fungi. Palladium nanoparticles (PdNPs) have brilliant catalytic, electronic, physical, mechanical and optical properties and have impressive potential for the development of novel photothermal, photoacoustic, antimicrobial and antitumor agents, gene/drug carriers, prodrug activators and biosensors [166]. Biosynthesized PdNPs have been found to possess more enhanced anticancer activities, as compared to other synthetic anticancer drugs [167]. However, the possibility of using the unique properties of palladium in various areas of biomedicine is still understudied and needs further research. To date, the mycosynthesis of this element in fungal cultures has remained extremely understudied, as compared to plants [167], and has been reported only in a few publications (Table 4).

Incubation of an *Agaricus bisporus* mushroom extract with palladium acetate resulted in the formation of PdNPs with anticancer, anti-inflammatory, antibacterial and antioxidant activities [168]. Porous anisotropic palladium nanoparticles were synthesized using an extract from the powder of the medicinal chaga mushroom (*I. obliquus*) [169]. The morphology of these nanoparticles could be controlled by changing the chaga extract concentration—with its increase, their rough surface morphology and porosity also increased. The properties of the obtained nanostructures showed their potential for biological-chemo-thermo tri-modal anticancer therapy.

PdNP synthesis using the baker’s yeast *Saccharomyces cerevisiae* biomass has also been reported. Saitoh and colleagues described the formation of crystalline PdNPs with diameters of 10–20 nm, deposited on the surfaces of the *S. cerevisiae* cells [171]. Other researchers have obtained hexagonal PdNPs using a dry yeast extract [170]. The synthesized PdNPs were found to be active toward the photocatalytic degradation of the azo dye.

### 2.5. Mycosynthesis of Copper Nanoparticles

Copper nanoparticles (CuNPs) have attracted attention owing to their optical, catalytic, mechanical, electrical and biomedical properties [172]. Biosynthesized CuNPs have antibacterial, antifungal, antiviral and anticancer properties and can be used in targeted drug delivery, cosmetic applications, catalysis, microelectronics, gas sensors, high-temperature superconductors, solar cells, as bactericide agents, wound dressings, biopesticides, in bioremediation, biodegradation and energy storage [172,173,174].

The mycosynthesis of CuNPs also remains poorly studied. An analysis of the literature showed that CuNPs produced by fungi are predominantly spherical in shape (Table 5).

Irregular spherical CuNPs of 5–25 size with antimicrobial activity against phytopathogens were obtained from a mycelial extract of *Trichoderma atroviride* [183]. Salvadori et al. found that *Trichoderma koningiopsis* can produce CuNPs [184]. Live, dead (autoclaved) and dried biomass of *T. koningiopsis* was used in the experiment. The dead biomass showed a higher capacity to adsorb copper metal ions than live and dried biomass and was used for further nanoparticle production. The resulting nanoparticles were predominantly spheric (average size of 87.5 nm) and were formed extracellularly. Similar results were shown for *Hypocrea lixii*, but the nanoparticles were smaller (average size of 24.5 nm) [180].

Cuevas et al. compared the CuNP mycosynthesis by a *Stereum hirsutum* extract using three different salts (CuSO_4_, Cu(NO_3_)_2_ and CuCl_2_) [182]. Nanoparticle biosynthesis in the presence of all copper salts demonstrated higher formation with 5 mM CuCl_2_ under alkaline conditions. The nanoparticles were mainly spherical (5 to 20 nm).

Copper nanospheres with their diameters ranging from 2 to 60 nm were formed on the surface of the *Aspergillus flavus* mycelium [176]. Noor et al. studied CuNP synthesis with *A. niger* [177]. The ability to mycosynthesize CuNPs was found in only one of the eight *A. niger* strains studied. The CuNPs were spherical and uniformly distributed without substantial agglomeration. Their size ranged from 5 to 100 nm. These nanoparticles showed anticancer, antimicrobial and antidiabetic activity. Nanoparticles 23–82 nm in size with a round to polygonal shape were obtained using an *Aspergillus versicolor* mycelial extract [178]. An antifungal study showed their potential antifungal activity against rotting plant pathogens.

Spherical CuNPs with excellent antibacterial, free-radical-scavenging and cytotoxic effects were obtained using an aqueous extract of *A. bisporus* fruit bodies [175]. CuNPs (40–65 nm in diameter) obtained with *Shizophyllum commune* biomass [181] exhibited antimicrobial and antibiofilm activity against human pathogens. *F. oxysporum* was found to leach copper from electronic waste composed of integrated circuits from obsolete and discarded electronic goods, forming nanoparticles [179], which opens the prospect of using myconanosynthesis in the bioremediation of electronic waste in order to recycle valuable metals.

### 2.6. Mycosynthesis of Iron Nanoparticles

Iron is one of the most abundant elements in the Earth’s crust that has been used by humankind for thousands of years, but only recently, with the development of nanotechnology, has it become a focus of interest in this new field of application. Iron nanoparticles (FeNPs) and iron-based nanomaterials are very important for the abatement of environmental pollution (degradation of organic dyes and other pollutants, heavy metal removal, wastewater treatment) and for use in biomedicine as antimicrobial agents [185,186]. So far, the production of FeNPs has been studied mainly in *Ascomycota* micromycetes (Table 6).

The table shows that mycogenic FeNPs are predominantly spherical in shape. For example, iron nanospheres approximately 100 nm in diameter with antimicrobial activity were obtained from a *P. florida* fruit body extract [192].

An *Aspergillus oryzae* extract was used to make 10–24.6 nm nanospheres [189]. Incubation of a *Penicillium oxalicum* mycelial extract with FeSO_4_ produced spherical nanoparticles with an average diameter of 140 nm, which effectively decolorized methylene blue dye [191]. FeNPs were also obtained using a cell-free filtrate extract of the *Rhizopus stolonifer* mycelium [194] and *Trichoderma* sp. [195].

Small cubic-shaped FeNPs with antibacterial activity against Gram-positive and Gram-negative bacteria were obtained by incubating an *A. alternata* mycelial extract with Fe(NO_3_)_3_ [187]. Other researchers obtained FeNPs using an *A. alternata* extract and FeSO_4_ as a precursor and found that the size and shape of the synthesized nanoparticles depended on the medium in which the fungal culture was grown [188]. The culture grown in a potato dextrose broth biosynthesized semi-oval polydisperse particles with size in the range of 20–40 nm, while fungi grown on the Czapek media produced particles with spheroid morphology of 10 to 80 nm. Six months after their production, 5 µm microparticles were formed from the mycosynthesized nanoparticles, possibly owing to the magnetic attraction of these materials.

Mazumdar and Haloi found that when a *Pleurotus* sp. mycelium was incubated with FeSO_4_, nanoparticles were synthesized both extra- and intracellularly [193]. A distinct layer of ferric nanoparticles was formed around the cells. The amount of nanoparticles inside the cells was significantly lower than outside. Nanoparticles synthesized with *F. oxysporum* biomass [190] had a size of 20–40 nm and possessed antimicrobial activity, but it was less pronounced than that of silver nanoparticles studied in the same work.

### 2.7. Mycosynthesis of Selenium Nanoparticles

In addition to metals, fungi have also been found to biosynthesize nanoparticles of metalloids, most notably selenium. Selenium is an essential element for humans, animals and microorganisms, but many of its compounds are highly toxic. Selenium nanoparticles (SeNPs) are of great interest owing to their lower toxicity than inorganic and organic forms of selenium and their biocompatibility, bioavailability and biomedical properties. Nano-selenium exhibits excellent antimicrobial, anticancer, antidiabetic, antiparasitic and antioxidant activities [196]. SeNPs can be used in targeted drug delivery, bioremediation, nanobiosensors, as a food supplement and in many other areas [197,198].

The ability of fungi to convert selenium from selenites, selenates and other compounds into elementary form to reduce their toxic effects has long been known to mycologists. For example, in as early as 1995, a number of fungal cultures were shown to reduce selenite to elementary selenium [199]. *Aspergillus funiculosus* and *Fusarium* sp. incubated with sodium selenite produced needle-like crystals of elementary selenium on the surfaces of hyphae and conidia. *A. funiculosus* also deposited electron-dense granules in vacuoles of selenite-treated fungi. However, SeNP mycosynthesis has received considerable attention only in recent years. So far, the biological synthesis of SeNPs has been detected in a fairly large number of fungal species and is best studied in microscopic ascomycetes (Table 7). As can be seen from the table, mycogenic SeNPs are mostly spherical in shape, and their diameter can vary widely. In some cultures, such as *A. alternata*, *Fusarium equiseti* and *Rhodotorula mucilaginosa*, the formation of selenium nanorods was also observed.

The ability to mycosynthesize SeNPs has been best studied in several species of *Penicillium*, *Aspergillus* and *Trichoderma*. For example, monodispersed selenium nanospheres with an average size of 24.65 nm exhibiting antibacterial activity were obtained using *P. chrysogenum* culture liquid and Na_2_SeO_3_ as a precursor [212]. Other researchers obtained molluscicidal and larvicidal SeNPs (44–78 nm) using a *P. chrysogenum* culture liquid [213]. El-Sayyad et al. developed a method to produce nanoparticles involving the incubation of a *P. chrysogenum* filtrate with Na_2_SeO_4_ following the application of gamma irradiation [214]. These nanospheres had an average diameter of 33.84 nm and exhibited antimicrobial and antibiofilm activities.

Amin et al. used another method to produce SeNPs involving the use of gamma radiation [215]. The spore suspension of *Penicillium citrinum* was exposed to different doses of gamma radiation, and SeNPs were then produced by an irradiated *P. citrinum*. Irradiation by gamma rays enhanced the mycosynthesis of SeNPs, and the size of the nanoparticles was dependent on the radiation dose.

Spherical SeNPs obtained with *Penicillium corylophilum* culture liquid had antimicrobial, cytotoxic and larvicidal activity against the mosquito vector of malaria [216]. Nanospheres with a diameter of 3–22 nm exhibiting antimicrobial, anticancer and catalytic activity were obtained using *Penicillium crustosum* culture liquid [217]. It was found that the presence of light is one of the influential parameters to promote these activities of SeNPs. Small selenium nanospheres with a wide range of biomedical activities, including antimicrobial activity against fungi, Gram-positive and Gram-negative bacteria and antioxidant and anticancer activity, were obtained using *Penicillium expansum* culture liquid [218].

Hussein et al. isolated several species of microscopic fungi with the ability to mycosynthesize SeNPs [203]. *Aspergillus quadrilineatus*, *Aspergillus ochraceusand* and *Aspergillus terreus* produced nanospheres of different sizes depending on the species, and *Fusarium equiseti* synthesized spherical and rod-shaped particles. The nanoparticles obtained had antibacterial, antifungal and antioxidant properties. Selenium nanospheres (average size of 47 nm) were also obtained using *A. terreus* culture liquid [204]. Selenium nanospheres mycosynthesized with *Aspergillus flavus* and *Candida albicans* culture liquid exhibited high antifungal activity showing inhibition of fungal growth in the presence of lower concentrations of nanoparticles than antifungal drugs [202].

Mycogenic selenium nanospheres synthesized from a *T. atroviride* extract had antifungal activity and also possessed the unique property of aggregating and binding to the zoospores of the phytopatogenic oomycete fungi *Phytophthora infestans* [221]. Spherical and pseudospherical SeNPs were synthesized by *Trichoderma* sp. on a medium with SeO_2_ [223]. The authors determined the optimal pH values, precursor concentration and application time for nanoparticle synthesis. Elementary selenium nanospheres (40–100 nm) with larvicidal activity were obtained using a *Trichoderma* sp. extract [224]. Hu et al. obtained irregularly spherical SeNPs using *T. harzianum* [222]. Many organic acids, sugars and their derivatives, such as heptonic acid, ferulate, fumaric acid and threonic acid, as well as glucose and mannitol, capped the selenium nanoparticles and played the role of stabilizing agents. Mycogenic nanoparticles inhibited the growth of pathogenic fungi better than traditionally produced SeNPs.

The fungi *T. harzianum*, *Aureobasidium pullulans*, *Mortierella humilis* and *Phoma glomerata* were able to grow on selenium-containing media resulting in the extensive precipitation of elementary selenium nanoparticles on fungal surfaces [205]. The average size of the *A. pullulans*-synthesized nanospheres was 60 nm, and that of *M. humilis*-synthesized nanoparticles was about 48 nm. Nanospheres with the size of 20–120 nm were formed when *A. pullulans* culture liquid was incubated with selenite [206]. Spherical nanoparticles (100–200 nm) formed on fungal surfaces and in the medium during the growth of *P. glomerata* with selenite [219]. 

Sarkar et al. reported on the synthesis of SeNPs with a spherical [200] or rod-like shape [201] using an *A. alternata* culture filtrate. When *F. oxysporum* biomass was incubated with selenious acid as a precursor, spherical selenium and selenium sulfide nanoparticles with their size between 34.32 and 231.98 nm were formed [207]. When the fungus *Mariannaea* sp. was grown in the presence of SeO_2_, intracellular and extracellular selenium nanospheres deposited on the cell wall and in the cytoplasmic region were formed [210]. The average size of the nanospheres was 45.19 nm for intracellular SeNPs and 212.65 nm for extracellular SeNPs.

Lian et al. studied SeNP production using cell-free extracts of a novel yeast, *Magnusiomyces ingens*, and showed that the pH, concentration of the selenium-containing compound SeO_2_ and protein content in the yeast extract could distinctly influence the formation and stabilization of SeNPs [209]. The SeNPs were almost quasispherical and spherical with a small number of irregular SeNPs. The diameter was mainly between 70 and 90 nm. Using the biomass of the yeast *Nematospora coryli*, selenium nanospheres with anti-*Candida* and anti-oxidant activities were obtained [211]. The aquatic yeast *R. mucilaginosa* synthesized SeNPs extracellularly and intracellularly [220]. Utilization of low selenite precursor concentrations (1–4 mM) resulted in the formation of spherical nanoparticles, and they formed rod-shaped structures at a higher precursor concentration (5 mM).

Compared to the cultures described above, the possibility of SeNP mycosynthesis using basidial macromycetes has been much less studied.

Vetchinkina et al. compared SeNP mycosynthesis by a number of edible and medicinal basidiomycetes using their mycelial extracts and culture liquids [41]. *A. arvensis*, *A. bisporus*, *G. lucidum* and *G. frondosa* produced selenium nanospheres whose size varied depending on the culture and method of their biosynthesis. In the case of *L. edodes* and *P. ostreatus*, nanospheres were fabricated using culture liquids while mycelial extracts produced irregularly spherical particles.

Living cultures *of G. lucidum*, *G. frondosa*, *L. edodes* and *P. ostreatus* also formed selenium nanospheres when grown on a medium with Na_2_SeO_3_ [208]. In *G. lucidum*, the diameter of the nanospheres was 20–50 nm; the other species synthesized larger particles (50–320 nm). Se^0^ nanoparticles were also formed when *L. edodes* was grown with the organic selenium compound but not with Na_2_SeO_4_ [225].

### 2.8. Mycosynthesis of Tellurium Nanoparticles

Tellurium is another metalloid that has recently attracted attention owing to its biogenic nanoparticles (TeNPs). Their photoconductive, thermoconductive, piezoelectric, non-linear optical, antioxidant, antimicrobial, anticancer, immunomodulating and cytotoxic properties, as well as their potential of being used in drug delivery, bioremediation and biorecovery, are of interest [226]. In fungi, TeNP formation is still very poorly studied (Table 8).

Biogenic TeNPs are typically rod-like or spherical. In *Phoma glomerata*, *Aureobasidium pullulans*, *Mortierella humilis* and *T. harzianum* cultures, the ability to form TeNPs intra- and extracellularly was found [205,219]. In *Phanerochaete chrysosporium*, the formation of needle-like particles (20–465 nm) of Te^0^ in the fungal hyphae was shown when incubated with TeO_3_^2-^ [229]. Tellurium nanospheres were obtained using culture liquids of *Aspergillus welwitschiae* [227] and *P. chrysogenum* [228].

## 3. Mechanisms of Fungi-Mediated Nanoparticle Biosynthesis

**Figure 1:**
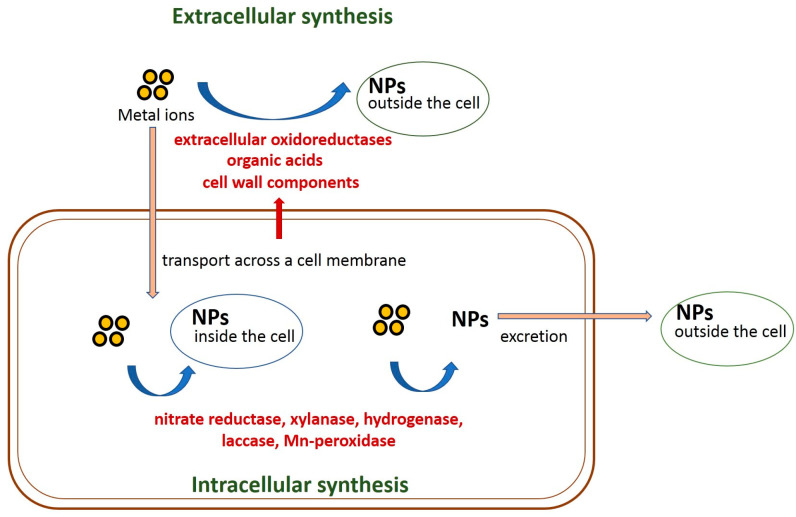
Schematic representation of fungi-mediated nanoparticle biosynthesis.

The process of nanoparticle formation by fungi can take place intra- and extracellularly under the action of enzymes and other biologically active molecules (Figure 1).

In recent years, more and more attention is paid to the study of the reduction mechanisms of various compounds by fungi, but not enough is known about particular compounds of the fungal secretome involved in the formation of nanoparticles. It has been found that laccase [146,230,231,232], Mn-peroxidase [146], tyrosinase [146] and ligninase [231] are involved in AuNP biosynthesis by fungi. Nitrate reductase [233], laccase [234,235] and xylanase [48] are involved in the AgNP mycosynthesis, while PtNP synthesis is catalyzed by hydrogenase [159,160]. The same culture can produce several enzymes catalyzing nanoparticle fabrication; for example, for *P. chrysosporium*, laccase and ligninase have been shown to be responsible for the extracellular and intracellular AuNP formation, respectively [231]. In addition to enzymatic myconanosynthesis, fungal peptides [236], polysaccharides [68,88,236] and phenolic compounds were found to participate in the reduction of various compounds and nanoparticle formation [86,141,237]. Thus, many researchers confirm that biological molecules such as polysaccharides, enzymes, proteins or peptides can be used for the synthesis and assembly of materials with nanoscale dimensions. Similar methods of the rational usage of biological processes could provide a new way for the development of nanotechnology.

## 4. Advantages of Fungi-Mediated Nanoparticle Synthesis and Prospects for Application of Mycogenic Nanoparticles

Among the variety of organisms capable of forming nanoparticles, fungi attract special attention. Owing to the unique properties of fungi, green fungal nanoparticle synthesis has several important advantages over the use of bacteria, plants and other organisms [40,238,239,240]. These advantages include:

- Active production of reducing and capping compounds;
- High activity of enzymes involved in the bioreduction of various compounds resulting in nanoparticle formation;
- Resistance to high concentrations of metals and metalloids;
- Ability to biofabricate large quantities of nanoparticles mostly extracellularly;
- High speed of nanoparticle formation;
- Simplicity of cultivation, nanoparticle downstream processing and scaling up;
- Safety for human health (when using edible and medicinal mushrooms);
- Ability to produce nanoparticles with complex medical properties (when using medicinal mushrooms).

Mycogenic nanoparticles have a wide range of biological activities that allow their use in many fields of medicine, agriculture and industry. Bactericidal, antibiofilm, fungicidal, antiviral, anticancer, anti-inflammatory, antioxidant, anticoagulant and thrombolytic properties of the mycogenic nanoparticles of gold, silver, platinum, palladium, copper, selenium and other elements allow their use in the therapy of various diseases, including cancer, Alzheimer’s disease, diabetes and cardiovascular and infectious diseases, as well as in wound healing [240,241,242]. It has been found that fungi-derived nanoparticles can effectively inhibit the growth of various pathogenic microorganisms [202], including drug-resistant pathogens [64,123]. The use of nanoparticles in combination with other antimicrobial agents increases their therapeutic effect and allows reducing the risk of the resistance development in pathogens, as well as restoring the activity of antibiotics that have lost their efficiency [243]. Of great interest is the use of medicinal fungi to produce nanoparticles with complex medical activity, which is achieved by combining the properties of the nanoparticles themselves and of the fungal metabolites acting as capping agents [169]. Activity of the nanoparticles against insect larvae and pupae spreading human diseases [53,216,244,245,246], as well as against pathogen vector mollusks [213,247], is also of interest in terms of the use of mycogenic AuNPs, AgNPs and SeNPs as an eco-friendly and cost-effective tool for disease biological control. Mycosynthesized nanoparticles have great potential for use as carriers in targeted drug delivery, for bioimaging and biolabeling, as sensors for optical and electronic devices and in the cosmetics, textile and food processing industries [240,248,249].

In agriculture, fungi-derived nanoparticles find application as nanopesticides and nanofertilizers, allowing one to reduce the use of more toxic agrochemicals [250,251,252,253]. Fungicidal, bactericidal, larvicidal and nematicidal activity found in many mycogenic nanoparticles [178,183,221,254,255,256,257] holds great promise for their use in the control of pests and phytopathogens, including pesticide-resistant ones. Another important area of application for mycogenic nanoparticles is mycoremediation [258]. The ability of fungi to utilize metal and metalloid compounds converting them into less toxic forms and accumulating them in the mycelium as nanoparticles is well known [259]. Due to this, fungi can be successfully used for metal and metalloid removal from soil and water and for their further recycling [184,260]. Immobilization of fungal biomass with nanoparticles allows one to obtain hybrid biosorbents for toxic element disposal [261]. The ability of mycogenic nanoparticles to degrade industrial and agricultural pollutants is of great interest. Various azo, diazo and metal-complex dyes are widely used in many industries; they get into water and soil in large quantities and pose a serious threat to the environment and human health, so recently the ability of fungi to produce nanoparticles with dye-degrading properties has been actively studied [262]. The dye-degrading activity found in mycogenic AuNPs [133,141], AgNPs [48], FeNPs [191] and PdNPs [170] makes them promising tools for eliminating industrial and municipal wastewater contamination by toxic dyes. Furthermore, fungi-derived nanoparticles have shown to be effective for pesticide removal [263] and for the treatment of wastewaters polluted with microbial contaminants [79].

## 5. Conclusions

The characteristics of mycogenic nanoparticles, including their shape, size, surface topography, homogeneity, resistance to aggregation and formation rate, can vary greatly for the same element. They depend on the physico-chemical parameters of the bioreduction process (chemical composition and concentration of the precursor, composition of the cultivation medium, pH, temperature, reaction time, presence of agitation and lighting, as well as additional influences such as microwave radiation and gamma radiation) and biological parameters (fungal species and strain, culture age, extract type and metabolites used).

**Figure 2:**
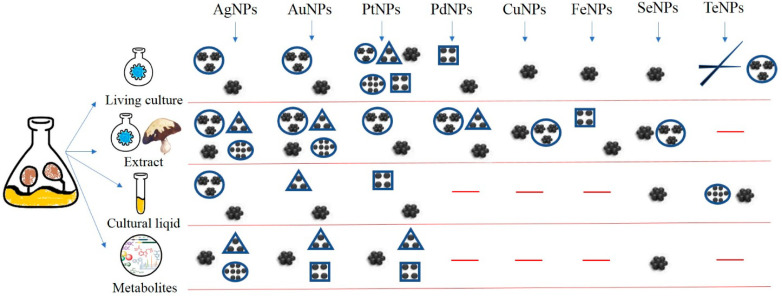
Mycosynthesis of various nanoparticles.

The ability to form nanoparticles has been found in many fungal species, predominantly belonging to the *Ascomycota* and *Basidiomycota*. Studies on the screening of various fungal cultures to identify the best nanoparticle producers show that different species and strains of the same species can vary greatly in nanosynthetic activity under the same conditions. The way in which fungal cultures are used to produce nanoparticles is also very important—whether in the form of living cultures grown on media with precursors, as filtered mycelial biomass, cell-free culture liquid, purified metabolites, extracts from a submerged mycelium, fruit bodies or other morphological formations. A summary of nanoparticle mycosynthesis is shown in Figure 2.

All these methods have their advantages and disadvantages. Living cultures growing on media with metal ions and metalloids can actively produce nanoparticles and accumulate them in the medium and inside their cells in very large quantities, but these particles require separation from the hyphae for their further use. Therefore, filtrates of culture liquids, extracts from undestroyed or destroyed mycelium and commercially purchased fruit bodies may be easier to use. Studies of nanoparticle mycosynthesis using enzymes and other compounds isolated from fungi can broaden the knowledge on the mechanisms of nanoparticle formation by fungi and are therefore of great importance for the development of fundamental science.

The fungi-mediated synthesis of elementary silver, gold and, to a lesser extent, selenium nanoparticles has now been studied in some detail and continues to be actively researched. Yet, other elements remain little explored or almost not at all in terms of myconanosynthesis. An important remaining task is the need to deepen and broaden our knowledge of fungi capable to biosynthesize nanoparticles of various chemical elements, search for new producers and optimize nanosynthesis processes for the efficient and controlled fabrication of particles with the desired properties. Green fungi-mediated nanoparticle synthesis is an eco-friendly and effective method that still needs further research.

## Figures and Tables

**Table 1:** Mycosynthesis of silver nanoparticles.

Species	Source	Precursor	Nanoparticles	References
*Agaricus arvensis*	Living culture	AgNO_3_	Spherical (10–20 nm)	[41]
	Cultural liquid	AgNO_3_	Irregular spherical (10–100 nm)	
	Mycelial extract	AgNO_3_	Spherical (1–10 nm)	
*Agaricus bisporus*	Living culture	AgNO_3_	Spherical (10–20 nm)	[41]
	Cultural liquid	AgNO_3_	Irregular spherical (10–100 nm)	
	Mycelial extract	AgNO_3_	Spherical (1–10 nm)	
*Agaricus bisporus*	Crude polysaccharide extract	AgNO_3_	Irregularly quasi-spherical (20–40 nm)	[88]
*Agaricus bisporus*	Fruit body extract	AgNO_3_	Face-centered cubic (average size of 43.9 nm)	[89]
*Agaricus bisporus*	Fruit body extract	AgNO_3_	Cubic (average size of 50.44 nm)	[90]
*Agaricus bisporus*	Fruit body extract	AgNO_3_	Spherical (average size of 16 nm)	[91]
*Agaricus brasiliensis*	Crude polysaccharide extract	AgNO_3_	Irregularly quasi-spherical (20–40 nm)	[88]
*Alternaria sp.*	Mycelial extract	AgNO_3_	Spherical (3–10 nm)	[47]
*Aspergillus niger*	Crude xylanase	AgNO_3_	Spherical, cylindrical, oval (15.21–77.49 nm)	[48]
*Auricularia polytricha*	Mycelial extract	AgNO_3_	Spherical (5–50 nm)	[92]
*Beauveria bassiana*	Mycelial extract	AgNO_3_	Triangular, circular, hexagonal (10–50 nm)	[50]
*Botryodiplodia theobromae*	Mycelial extract	AgNO_3_	66.75–111.23 nm	[52]
	Mycelial biomass	AgNO_3_	62.77–103 nm	
*Flammulina velutipes*	Fungal extract	AgNO_3_	Spherical (average size of 21.4 nm)	[97]
*Flammulina velutipes*	Fruit body extract	AgNO_3_	Spherical (average size of 22 nm)	[98]
*Fomes fomentarius*	Fruit body extract	AgNO_3_	Spherical (10–20 nm)	[99]
*Fomitopsis pinicola*	Fruit body extract	AgNO_3_	Spherical (10–30 nm)	[100]
*Ganoderma applanatum*	Fruit body extract	AgNO_3_	Spherical (average size of 58.77 nm)	[102]
*Ganoderma lucidum*	Living culture	AgNO_3_	Spherical (10–20 nm)	[41]
	Cultural liquid	AgNO_3_	Irregular spherical (10–100 nm)	
	Mycelial extract	AgNO_3_	Spherical (1–10 nm)	
	Fruit body extract	AgNO_3_	Near-cubic (20–200 nm)	
*Ganoderma lucidum*	Fungal extract	AgNO_3_	Spherical (23–58 nm)	[123]
*Ganoderma lucidum*	Fruit body extract	AgNO_3_	Spherical (15–22 nm)	[124]
*Ganoderma lucidum*	Fruit body extract	AgNO_3_	Spherical (average size of 11.38 nm)	[125]
*Ganoderma sessile*	Mycelial extract	AgNO_3_	Quasi-spherical (average size of 5.4 or 8.9 nm depending on the extraction method)	[82]
*Ganoderma sessiliforme*	Fruit body extract	AgNO_3_	Spherical (average size of 45 nm)	[101]
*Grifola frondosa*	Living culture	AgNO_3_	Spherical (10–20 nm)	[41]
	Cultural liquid	AgNO_3_	Irregular spherical (10–100 nm)	
	Mycelial extract	AgNO_3_	Spherical (1–10 nm)	
*Helvella leucopus*	Fruit body extract	AgNO_3_	Spherical (80–100 nm), aggregated	[61]
*Lactarius piperatus*	Fruit body extract	AgNO_3_	Spherical (average size of 49 nm)	[105]
*Lentinus edodes*	Living culture	AgNO_3_	Spherical (10–20 nm)	[41]
	Cultural liquid	AgNO_3_	Irregular spherical (10–100 nm), spherical conglomerates 50–250)	
	Mycelial extract	AgNO_3_	Spherical (1–10 nm)	
*Lentinus tuber-regium*	Fruit body extract	AgNO_3_	Spherical (5–35 nm)	[107]
*Penicillium citrinum*	Mycelial extract	AgNO_3_	Spherical (2–5 nm)	[67]
*Penicillium cyclopium*	Mycelial biomass	AgNO_3_	Mostly irregular (12–25 nm)	[68]
*Penicillium janthinellum*	Mycelial extract	AgNO_3_	Spherical (1–30 nm)	[69]
*Penicillium oxalicum*	Mycelial extract	AgNO_3_	Spherical (60–80 nm)	[70]
*Penicillium oxalicum*	Mycelial extract	AgNO_3_	Spherical (average size of 52.26 nm)	[71]
*Penicillium polonicum*	Mycelial extract	AgNO_3_	Mostly spherical (10–15 nm), hexagonal, polyhedral (above 30 nm)	[72]
*Phaenerochaete chrysosporium*	Mycelial extract	AgNO_3_	Spherical, oval (34–90 nm)	[109]
*Phellinus linteus*	Crude polysaccharide extract	AgNO_3_	Irregularly quasi-spherical (20–40 nm)	[88]
*Picoa* sp.	Fruit body extract	AgNO_3_	Irregular (average size of 19.5 nm)	[76]
*Pleurotus djamor*	Fruit body extract	AgNO_3_	Spherical (average size of 55.76 nm)	[114]
*Pleurotus eryngii*	Fruit body extract	AgNO_3_	Spherical (average size of 18.45 nm)	[112]
*Pleurotus florida*	Fruit body extract	AgNO_3_	Spherical (average size of 10 nm)	[111]
*Pleurotus ostreatus*	Living culture	AgNO_3_	Spherical (10–20 nm)	[41]
	Cultural liquid	AgNO_3_	Irregular spherical (10–100 nm)	
	Mycelial extract	AgNO_3_	Spherical (1–10 nm)	
*Pleurotus ostreatus*	Fruit body extract	AgNO_3_	Spherical, hexagonal (18–82 nm)	[126]
*Pleurotus ostreatus*	Fruit body extract	AgNO_3_	Spherical (average size of 28.44 nm)	[114]
*Pleurotus sajor caju*	Fruit body extract	AgNO_3_	Spherical (11–44 nm)	[127]
*Pleurotus sajor caju*	Fruit body extract	AgNO_3_	Spherical (average size of 15–20 nm)	[113]
*Tirmania* sp.	Fruit body extract	AgNO_3_	Irregular, spherical (average size of 72 nm)	[81]
*Trametes trogii*	Mycelial extract	AgNO_3_	Mostly spherical (5–65 nm)	[118]
*Trichoderma atroviride*	Mycelial extract	AgNO_3_	15–25 nm	[84]
*Trichoderma atroviride*	Cultural liquid	AgNO_3_	Spherical (20–30 nm)	[42]
	Mycelial extract	AgNO_3_	Spherical (15–35 nm)	
*Trichoderma harzianum*	Mycelial extract	AgNO_3_	Spherical (10–25 nm)	[83]
*Trichoderma harzianum*	Mycelial extract	AgNO_3_	Quasi-spherical (average size of 9.6 or 19.1 nm depending on the extraction method)	[82]
*Trichoderma longibrachiatum*	Crude xylanase	AgNO_3_	Spherical, cylindrical, oval (15.21–77.49 nm)	[48]
*Trichoderma longibrachiatum*	Cultural liquid	AgNO_3_	Spherical (5–15 nm)	[42]
	Mycelial extract	AgNO_3_	Spherical (10–25 nm)	

**Table 2:** Mycosynthesis of gold nanoparticles.

Species	Source	Precursor	Nanoparticles	References
*Agaricus arvensis*	Living culture	HAuCl_4_	Spherical (5–50 nm)	[41]
	Cultural liquid	HAuCl_4_	Spherical (2–10 nm)	
	Mycelial extract	HAuCl_4_	Irregular spherical (25–20 nm)	
*Agaricus bisporus*	Fruit body extract	HAuCl_4_	Spherical (average size of 25 nm)	[132]
*Agaricus bisporus*	Living culture	HAuCl_4_	Spherical (5–50 nm)	[41]
	Cultural liquid	HAuCl_4_	Spherical (2–10 nm)	
	Mycelial extract	HAuCl_4_	Spherical (10–50 nm), hexagonal, tetragonal, triangular (30–100 nm)	
*Agaricus bisporus*	Fruit body extract	HAuCl_4_	Oval, spherical, drum-like, hexagonal, triangular (average size of 53 nm)	[133]
*Agaricus bisporus*	Fruit body extract	HAuCl_4_	Spherical (10–50 nm)	[134]
*Alternaria* spp.	Fungal extract	HAuCl_4_	Triangular, circular (average size of 28 nm)	[135]
*Cantharellus* sp.	Fungal extract	HAuCl_4_	Spherical (average size of 60.6 nm)	[136]
*Coprinus comatus*	Fruit body extract	HAuCl_4_	Face-centered cubic (average size of 17.39 nm)	[137]
*Flammulina velutipes*	Fruit body extract	HAuCl_4_	Triangular, spherical, irregular (average size of 74.32 nm)	[138]
*Fusarium oxysporum*	Cultural liquid	HAuCl_4_	Spherical, hexagonal (22–30 nm)	[139]
*Fusarium solani*	Biomass extract	HAuCl_4_	Needle and flower-like structures with spindle shape (40–45 nm)	[140]
*Ganoderma applanatum*	Isolated phenolic compounds	HAuCl_4_	Face-centered cubic (average size of 18.70 nm)	[141]
*Ganoderma lucidum*	Living culture	HAuCl_4_	Spherical (5–50 nm)	[41]
	Cultural liquid	HAuCl_4_	Spherical (5–60 nm)	
	Mycelial extract	HAuCl_4_	Spherical (10–50 nm), hexagonal, tetragonal, triangular (30–100 nm)	
*Ganoderma lucidum*	Fruit body extract	HAuCl_4_	Spherical, oval, irregular (1–100 nm)	[142]
*Grifola frondosa*	Living culture	HAuCl_4_	Spherical (5–50 nm)	[41]
	Cultural liquid	HAuCl_4_	Spherical (2–10 nm)	
	Mycelial extract	HAuCl_4_	Spherical (10–50 nm), hexagonal, tetragonal, triangular (30–100 nm)	
*Inonotus obliquus*	Fruit body extract	HAuCl_4_	Mostly spherical (below 20 nm)	[143]
*Laetiporus versisporus*	Fruit body extract	HAuCl_4_	Spherical (average size of 10 nm)	[144]
*Lentinus edodes*	Fruit body extract	HAuCl_4_	Triangular, hexagonal, spherical, irregular (average size of 72 nm)	[145]
*Lentinus edodes*	Living culture	HAuCl_4_	Spherical (5–50 nm)	[41,146]
	Cultural liquid	HAuCl_4_	Spherical (2–20 nm)	
	Mycelial extract	HAuCl_4_	Spherical (10–50 nm), hexagonal, tetragonal, triangular (30–200 nm)	
	Intracellular Mn-peroxidase	HAuCl_4_	Spherical (2–20 nm)	
	Intracellular laccases and tyrosinases	HAuCl_4_	Irregular spherical, triangular, tetrahedral (5–120 nm)	
*Morchella esculenta*	Fruit body extract	HAuCl_4_	Face-centered cubic (average size of 16.51 nm)	[147]
*Penicillium janthinellum*	Mycelial extract	HAuCl_4_	Spherical (1–40 nm)	[69]
*Phoma* sp.	Mycelial biomass	HAuCl_4_	Spherical (10–100 nm)	[148]
*Pleurotus ostreatus*	Living culture	HAuCl_4_	Spherical (5–50 nm)	[41]
	Cultural liquid	HAuCl_4_	Spherical (2–20 nm)	
	Mycelial extract	HAuCl_4_	Spherical (10–50 nm), hexagonal, tetragonal, triangular (30–200 nm)	
*Pleurotus sajor-caju*	Fruit body extract	HAuCl_4_	Spherical (average size of 16–18 nm)	[113]
*Trichoderma hamatum*	Mycelial extract	HAuCl_4_	Spherical, pentagonal, hexagonal (5–30 nm)	[149]
*Trichoderma harzianum*	Mycelial biomass	HAuCl_4_	Spherical (below 30 nm)	[150]
*Tricholoma crassum*	Mycelial extract	HAuCl_4_	Circular, rhomboid (5 nm or less), hexagonal, cubic, triangular (4.36–22.94 nm)	[151]

**Table 3:** Mycosynthesis of platinum nanoparticles.

Species	Source	Precursor	Nanoparticles	References
*Alternaria alternata*	Cultural liquid	H_2_PtCl_6_	Irregular (50–315)	[157]
*Fusarium oxysporum*	Mycelial biomass	H_2_PtCl_6_	Hexagonal, pentagonal, circular, square, rectangular (10–100 nm)	[158]
*Fusarium oxysporum*	Purified mycelial enzyme	PtCl_2_	Rectangular, triangular (100–180 nm)	[159]
	Purified mycelial enzyme	H_2_PtCl_6_	Spherical (100–140 nm)	
*Fusarium oxysporum*	Mycelial extract	H_2_PtCl_6_	Irregular (30–40 nm)	[160]
	Purified mycelial enzyme	H_2_PtCl_6_	Circular, triangular, pentagonal, hexagonal, often as nanoplates (40–60 nm)	
*Fusarium oxysporum*	Mycelial biomass	H_2_PtCl_6_	Spherical (15–30 nm)	[161]
*Fusarium oxysporum*	Cultural liquid	H_2_PtCl_6_	Face-centered cubic (average size of 25 nm)	[162]
*Neurospora crassa*	Mycelial biomass	H_2_PtCl_6_	Quazi-spherical single PtNPs (4–35 nm) and spherical nanoaggregates (20–110 nm)	[163]
	Mycelial extract	H_2_PtCl_6_	Spherical nanoaggregates (17–76 nm), containing individual single crystals 2–3 nm in diameter	
*Penicillium chrysogenum*	Cultural liquid	H_2_PtCl_6_	Spherical (5–40 nm)	[164]
*Saccharomyces boulardii*	Cell free extract	H_2_PtCl_6_	Spherical (80–150 nm)	[165]

**Table 4:** Mycosynthesis of palladium nanoparticles.

Species	Source	Precursor	Nanoparticles	References
*Agaricus bisporus*	Mushroom extract	[Pd(OAc)_2_]_n_	Triangular and spherical (13–18 nm)	[168]
*Inonotus obliquus*	Fruit body powder extract	PdCl_4_^2−^	Porous spherical	[169]
*Saccharomyces cerevisiae*	Biomass extract	[Pd(OAc)_2_]_n_	Hexagonal (average size of 32 nm), agglomerated	[170]
*Saccharomyces cerevisiae*	Biomass	Na_2_PdCl_4_	Spherical (10–20 nm)	[171]

**Table 5:** Mycosynthesis of copper nanoparticles.

Species	Source	Precursor	Nanoparticles	References
*Agaricus bisporus*	Fruit body extract	Cu(NO_3_)_2_	Spherical (10–60 nm)	[175]
*Aspergillus flavus*	Mycelial biomass	CuSO_4_	Spherical (2–60 nm)	[176]
*Aspergillus niger*	Mycelial extract	CuSO_4_	Spherical (5–100 nm)	[177]
*Aspergillus versicolor*	Mycelial extract	CuSO_4_	Spherical, polygonal (23–82 nm)	[178]
*Fusarium oxysporum*	Mycelial biomass	Copper-containing waste	Spherical (93–115 nm)	[179]
*Hypocrea lixii*	Mycelial biomass	CuCl_2_	Spherical (average size of 24.5 nm)	[180]
*Shizophyllum commune*	Mycelial biomass	CuCl_2_	Spherical (40–65 nm)	[181]
*Stereum hirsutum*	Mycelial extract	CuCl_2_	Spherical (5–20 nm)	[182]
*Trichoderma atroviride*	Mycelial extract	CuSO_4_	Irregular spherical (5–25 nm)	[183]
*Trichoderma koningiopsis*	Mycelial biomass	CuCl_2_	Spherical (average size of 87.5 nm)	[184]

**Table 6:** Mycosynthesis of iron nanoparticles.

Species	Source	Precursor	Nanoparticles	References
*Alternaria alternata*	Mycelial extract	Fe(NO_3_)_3_	Cubic (average size of 9 nm)	[187]
*Alternaria alternata*	Mycelial extract	FeSO_4_	Semi-oval (20–40 nm)/spherical (10–80 nm)	[188]
*Aspergillus oryzae*	Mycelial extract	FeCl_3_	Spherical (10–24.6 nm)	[189]
*Fusarium oxysporum*	Mycelial biomass	K_3_Fe(CN)_6_	Spherical (20–40 nm)	[190]
		K_4_Fe(CN)_6_		
*Penicillium oxalicum*	Mycelial extract	FeSO_4_	Spherical (average size of 140 nm)	[191]
*Pleurotus florida*	Fruit body extract	FeCl_3_	Spherical (100 nm)	[192]
*Pleurotus* sp.	Mycelial biomass	FeSO_4_	–	[193]
*Rhizopus stolonifer*	Mycelial extract	FeCl_3_	–	[194]
*Trichoderma* sp.	Mycelial extract	FeCl_3_	–	[195]

**Table 7:** Mycosynthesis of selenium nanoparticles.

Species	Source	Precursor	Nanoparticles	References
*Agaricus arvensis*	Cultural liquid	Na_2_SeO_3_	Spherical (150–550 nm)	[41,153]
	Mycelial extract	Na_2_SeO_3_	Spherical (100–250 nm)	
*Agaricus bisporus*	Cultural liquid	Na_2_SeO_3_	Spherical (100–250 nm)	[41,153]
	Mycelial extract	Na_2_SeO_3_	Spherical (40–140 nm)	
*Alternaria alternata*	Cultural liquid	Na_2_SeO_4_	Spherical (30–150 nm)	[200]
*Alternaria alternata*	Cultural liquid	Na_2_SeO_4_	Nanorods (200–800 nm in length, 50–70 nm in width)	[201]
*Aspergillus flavus*	Cultural liquid	Na_2_SeO_4_	Spherical (average size of 51.5 nm)	[202]
*Aspergillus ochraceus*	Living culture	Na_2_SeO_3_	Spherical (average size of 45.22 nm)	[203]
*Aspergillus quadrilineatus*	Living culture	Na_2_SeO_3_	Spherical (average size of 55.37 nm)	[203]]
*Aspergillus terreus*	Cultural liquid	Se^4+^ ions solution	Spherical (average size of 47 nm)	[204]
*Aspergillus terreus*	Living culture	Na_2_SeO_3_	Spherical (average size of 30.98 nm)	[203]
*Aureobasidium pullulans*	Living culture	Na_2_SeO_3_	Spherical (average size of 60 nm)	[205]
*Aureobasidium pullulans*	Cultural liquid	Na_2_SeO_3_	Spherical (20–120 nm)	[206]
*Candida albicans*	Cultural liquid	Na_2_SeO_4_	Spherical (average size of 64 nm)	[202]
*Fusarium equiseti*	Living culture	Na_2_SeO_3_	Spherical and rod-shaped (average size of 30.11 nm)	[203]
*Fusarium oxysporum*	Biomass	H_2_SeO_3_	Spherical (34.32–231.98 nm)	[207]
*Ganoderma lucidum*	Living culture	Na_2_SeO_3_	Spherical (20–50 nm)	[208]
*Ganoderma lucidum*	Cultural liquid	Na_2_SeO_3_	Spherical (20–50 nm)	[41]
	Mycelial extract	Na_2_SeO_3_	Spherical (100–300 nm)	
*Grifola frondosa*	Living culture	Na_2_SeO_3_	Spherical (50–320 nm)	[208]
*Grifola frondosa*	Cultural liquid	Na_2_SeO_3_	Spherical (20–50 nm)	[41]
	Mycelial extract	Na_2_SeO_3_	Spherical (100–300 nm)	
*Lentinus edodes*	Living culture	Na_2_SeO_3_	Spherical (50–320 nm)	[208]
*Lentinus edodes*	Cultural liquid	Na_2_SeO_3_	Spherical (50–150 nm)	[41]
	Mycelial extract	Na_2_SeO_3_	Irregular spherical (50–150 nm)	
*Magnusiomyces ingens*	Biomass extract	SeO_2_	Spherical, quasi-spherical (70–90 nm)	[209]
*Mariannaea sp.*	Living culture	SeO_2_	Spherical (average size of 45.19/212.65 nm depending on the nanoparticle location)	[210]
*Mortierella humilis*	Living culture	Na_2_SeO_3_	Spherical (average size of 48 nm)	[205]
*Nematospora coryli*	Biomass	Na_2_SeO_3_	Spherical (50–250 nm)	[211]
*Penicillium chrysogenum*	Cultural liquid	Na_2_SeO_3_	Spherical (average size of 24.65 nm)	[212]
*Penicillium chrysogenum*	Cultural liquid	Na_2_SeO_3_	Spherical (44–78 nm)	[213]
*Penicillium chrysogenum*	Cultural liquid	Na_2_SeO_4_	Spherical (average size of 33.84 nm)	[214]
*Penicillium citrinum*	Biomass	HNaO_3_Se	Spherical (various sizes depending on the conditions)	[215]
*Penicillium corylophilum*	Cultural liquid	Na_2_SeO_3_	Spherical (29.1–48.9 nm)	[216]
*Penicillium crustosum*	Cultural liquid	Na_2_SeO_3_	Spherical (3–22 nm)	[217]
*Penicillium expansum*	Cultural liquid	SeO_2_	Spherical (4–12.7 nm)	[218]
*Phoma glomerata*	Living culture	Na_2_SeO_3_	Spherical (100–200 nm)	[219]
*Pleurotus ostreatus*	Living culture	Na_2_SeO_3_	Spherical (50–320 nm)	[208]
*Pleurotus ostreatus*	Cultural liquid	Na_2_SeO_3_	Spherical (50–150 nm)	[41]
	Mycelial extract	Na_2_SeO_3_	Irregular spherical (50–150 nm)	
*Rhodotorula mucilaginosa*	Biomass	Na_2_SeO_3_	Spherical, rod-shaped (83–478 nm depending on the precursor concentration)	[220]
*Trichoderma atroviride*	Mycelial extract	Na_2_SeO_3_	Spherical (60.48–123.16 nm)	[221]
*Trichoderma harzianum*	Mycelial extract	Na_2_SeO_3_	Irregular (average size of 60 nm)	[222]
*Trichoderma* sp.	Living culture	SeO_2_	Spherical, pseudo-spherical (20–220 nm)	[223]
*Trichoderma* sp.	Mycelial extract	–	Spherical (40–100 nm)	[224]

**Table 8:** Mycosynthesis of tellurium nanoparticles.

Species	Source	Precursor	Nanoparticles	References
*Aspergillus welwitschiae*	Cultural liquid	K_2_TeO_3_	Oval to spherical (60.80 nm)	[227]
*Aureobasidium pullulans*	Living culture	Na_2_TeO_3_	Granular	[205]
*Mortierella humilis*	Living culture	Na_2_TeO_3_	Granular	[205]
*Penicillium chrysogenum*	Cultural liquid	K_2_TeO_3_	Spherical (average size of 50.16 nm)	[228]
*Phanerochaete chrysosporium*	Living culture	K_2_TeO_3_	Needles (20–465 nm)	[229]
*Phoma glomerata*	Living culture	Na_2_TeO_3_	Pillars, needles	[205]
*Phoma glomerata*	Living culture	Na_2_TeO_3_	Rods (10–80 nm)	[219]
*Trichoderma harzianum*	Living culture	Na_2_TeO_3_	Pillars, needles, agglomerated rods	[205]

## Data Availability

Not applicable.
